# Comparing Building and Neighborhood-Scale Variability of CO_2_ and O_3_ to Inform Deployment Considerations for Low-Cost Sensor System Use

**DOI:** 10.3390/s18051349

**Published:** 2018-04-26

**Authors:** Ashley Collier-Oxandale, Evan Coffey, Jacob Thorson, Jill Johnston, Michael Hannigan

**Affiliations:** 1 Environmental Engineering, University of Colorado Boulder, Boulder, CO 80309, USA; 2 Mechanical Engineering, University of Colorado Boulder, Boulder, CO 80309, USA; evan.coffey@colorado.edu (E.C.); jacob.thorson@colorado.edu (J.T.); hannigan@colorado.edu (M.H.); 3 Preventive Medicine, University of Southern California, Los Angeles, CA 90089, USA; jillj@usc.edu

**Keywords:** low-cost sensors, gas-phase pollutants, air quality, spatial variability, best-practices, citizen science

## Abstract

The increased use of low-cost air quality sensor systems, particularly by communities, calls for the further development of best-practices to ensure these systems collect usable data. One area identified as requiring more attention is that of deployment logistics, that is, how to select deployment sites and how to strategically place sensors at these sites. Given that sensors are often placed at homes and businesses, ideal placement is not always possible. Considerations such as convenience, access, aesthetics, and safety are also important. To explore this issue, we placed multiple sensor systems at an existing field site allowing us to examine both neighborhood-level and building-level variability during a concurrent period for CO_2_ (a primary pollutant) and O_3_ (a secondary pollutant). In line with previous studies, we found that local and transported emissions as well as thermal differences in sensor systems drive variability, particularly for high-time resolution data. While this level of variability is unlikely to affect data on larger averaging scales, this variability could impact analysis if the user is interested in high-time resolution or examining local sources. However, with thoughtful placement and thorough documentation, high-time resolution data at the neighborhood level has the potential to provide us with entirely new information on local air quality trends and emissions.

## 1. Introduction and Background

As research into and the use of low-cost air quality sensor systems continues to expand there is great potential for this technology to support community-level investigations. Furthermore, given the nature of these sensor systems, such investigations provide data with increased resolution on both temporal and spatial scales. Ideally, such sensor systems offer greater insight into personal exposure [1], small-scale variability [2], and local emission sources or potential ‘hot spots’ [3]. One of the barriers to widespread sensor use has been concerns over data quality and reliability. There is a growing body of research demonstrating the ability of sensors to quantify pollutants at levels relevant to ambient investigations [4,5,6,7]. However, other issues have received less attention, for example, strategies for siting low-cost sensors. Sensor deployment and siting considerations are particularly important because while it is sometimes possible to re-analyze or re-quantify sensor data as new techniques become available, it is rarely possible to re-collect data as environmental conditions and emissions impacting a site are dynamic in nature. Careful consideration prior to and documentation of the sensor siting process could not only aid in data processing and interpretation, but also help to ensure the collection of useful and relevant data.

Previous studies have demonstrated that pollutant variability can exist on small spatial scales utilizing six-minute or hourly averaged data. Variability has been observed across a street or within a few hundred meters, especially in more complex urban environments [8,9]. Therefore, it is reasonable to expect that where the sensor is placed on a building could influence the data collected. While there are strict guidelines for siting a Federal Equivalent Method/Federal Reference Method (FEM/FRM) monitor, existing guidelines may not transfer well as the objectives of a community using sensors may differ from the purpose of a typical FRM/FEM instrument. A community group may wish to compare local emission sources or understand potential exposures at a neighborhood level, as opposed to characterize regional air quality. Further complicating the matter, siting a sensor system at a home or business can be challenging as convenience, safety, and aesthetics are all factors in the decision rather than simply the most ideal placement for accurate measurements.

A recent study in New Zealand thoroughly examined specific aspects of this question by determining the intra-site variability of paired O_3_ sensors and the impact of siting conditions/type on the overall dataset [10]. Miskell and colleagues found that most factors examined, such as placement at a site (i.e., on a wall, balcony, or roof), land coverage beneath the instrument, or land-use designation at the site, had little impact on the observed intra-site variability. Two factors–exposure to direct sunlight likely causing temperature differentials between paired sensors and local emission events–resulted in the greatest intra-site variability [10]. The team concluded that networks of O_3_ monitors set up by citizen scientists can supplement existing reference networks and provide new information, as limited variability was introduced due to siting choices and this variability was minimal over typical reporting scales (e.g., hourly or 8-hour averaged data) [10].

This study by Miskell and colleagues provided a comprehensive example of how sensor systems can support existing monitoring networks for O_3_ and the impacts of siting choices in this context. However, it is possible that communities may wish to use sensors for the collection of high-time resolution data on smaller spatial scales rather than the larger averaging times and regional scales studied by Miskell and colleagues. To explore the impact of siting choices in this alternate context we undertook a small case study during a larger deployment of sensor systems in Los Angeles, CA, USA. We added four additional sensor systems to one sampling site to observe the variability across several sensors on one building. We compared this building-level variability to the neighborhood-level variability. This analysis includes data from both metal oxide O_3_ sensors and non-dispersive infrared CO_2_ sensors–providing the opportunity to examine a primary and a secondary pollutant. The differing spatial scales (neighborhood vs. regional) and higher temporal resolution (utilizing primarily minute-median data) as well as the addition of CO_2_ data offers a small, complementary dataset providing additional information to inform recommendations for siting practices.

Furthermore, while there currently exist several valuable resources contributing best practices and supporting community-based investigations using low-costs sensors, such as the US EPA’s Air Sensor Guidebook [11], South Coast Air Quality Management District’s Sensor Performance and Evaluation Center [12], and the Environmental Defense Funds Air Sensor Work Group [13], additional case studies examining the questions of best-practices in different contexts will support the development of recommendations appropriate for the variety of uses likely to emerge. This need for more standards to guide all aspects of sensor use from planning to deployment to data analysis and interpretation has been cited as critical by academic, community-based, and regulatory researchers [14].

## 2. Materials & Methods

### 2.1. Deployment Overview (Sensor Systems, Siting, and Timeline)

**Figure 1:**
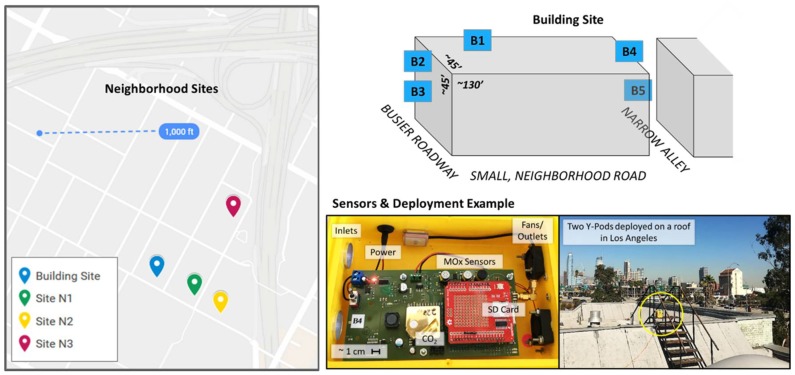
The map (**left**) indicates the sampling sites relevant to this paper, the diagram (**top right**) indicates where sensor systems were deployed at the Building Site, and the photos (**bottom right**) show the inside of a Y-Pod and a deployed Y-Pod.

The sensor systems utilized for this study, called Y-Pods (Hannigan Lab at CU Boulder, Boulder, CO, USA), contain several gas-phase and environmental sensors. This analysis utilizes data from the SGX (Corcelles-Cormondreche, Switzerland, formerly e2v) metal oxide semiconductor O_3_ sensors (model MiCS-2611) and ELT non-dispersive infrared CO_2_ sensors (model S-300) as well as data from environmental sensors (i.e., temperature and relative humidity). These sensor systems, or similar ones (e.g., the U-Pod, predecessor to the Y-Pod) operating the same sensors, have been used in prior sensor quantification and spatial variability studies [2,15,16,17,18]. Figure 1 includes a photo of the interior of a Y-Pod and an example of two deployed Y-Pods. The Y-Pods, and all previous iterations, include a fan to drive active air flow resulting in multiple air exchanges per minute. The observations presented here would likely need to be re-evaluated for a system relying on passive flow. More information on signal processing and sensor quantification is available below in Section 2.2.

As previously mentioned, this study was integrated into a larger field deployment in Los Angeles allowing us to leverage one of the existing study sites and ongoing sensor calibration efforts. The study area is primarily high density residential with schools and some businesses nearby. In addition to local traffic and businesses (such as restaurants) other emission sources include two major highways to the North and East of the sampling area. The diagram in Figure 1 illustrates where the Y-Pods (B2, B3, B4, and B5) were added to the building site (main sensor system–B1). Note, the placements vary with respect to elevation and proximity to obstructions. Two Y-Pods were placed on the front of the building on a fire escape, two and three stories off the ground, and 6–12″ from the side of the building. The fire escapes at the front and back of the building are both constructed of metal and allow for free airflow through and around the structures. The main Y-Pod was elevated on the roof, on top of a structure housing the stairs, close to the front of the building, and with no obstructions on any sides. The fourth and fifth Y-Pods were placed at the back of the building on another fire escape, one at the roof-level and the other three stories off the ground, again 6–12″ from the side of the building. The back of the building is obstructed by a narrow alley that does not allow through-traffic; the lack of access to representative air flow makes the placement of B5 the least “ideal”.

Figure 1 also illustrates the location of several other neighborhood sites from which data was used in this analysis (N1, N2, and N3). These sensor systems were deployed on a relatively small scale with the furthest distance between any two neighborhoods sites being less than 1000 ft. It is important to note that the placement of N1, N2, and N3 at their respective sites also introduces some added variability as these placements differed site to site. The Y-Pod placement for N1 was most similar to B3 on a large second story balcony, on the side of a building open to the road. The Y-Pod placement for N2 was also most similar to B3–at the front of the building, on the street side, but set back by a small yard/driveway and lower in elevation (~10 ft off the ground). The Y-Pod placement for N3 was most similar to B1, placed on the roof of a multi-family residence.

**Figure 2:**
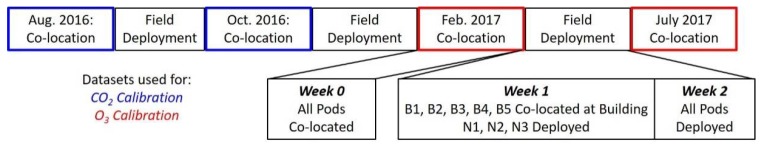
Timeline showing when co-location of sensors with reference instruments occurred and when deployments to field sites occurred.

This study relies on comparing co-located sensor data with spatially deployed sensor data, therefore we limited the data utilized to match the lengths of our co-located datasets meaning approximately three weeks of data were included in the analysis. Figure 2 shows the timeline of long-term sensor use, including time periods of co-location and periods of field deployment. The co-location of all sensor systems prior to the field deployment was used to understand neighborhood variability; this co-located time period is referred to as Week 0. The Week 0 co-location occurred in a different part of Los Angeles at a regulatory monitoring site; this site is described in greater detail below in Section 2.2.2. For the first week of the building-scale variability study, the building Y-Pods (B2, B3, B4, and B5) were co-located with B1–this is referred to as Week 1. During this period the neighborhood Y-Pods (N1, N2, and N3) were already deployed to their field sites. Immediately following the first week of the field deployment the sensor systems were separated to their respective locations on the building and this is referred to as Week 2. The data from Week 2 was designated as the deployed dataset for both the neighborhood sites and the building sites. 

### 2.2. Signal Processing and Sensor Quantification

Sensor signals were saved to a text file on a micro-SD card on the Y-Pod every 6–25 s, depending on the programming. As some of the metal oxide sensors used here require a warm-up period, the first half hour of data after a pod has been powered off for half an hour or more was removed. Minute-medians were computed; using medians instead of averages removes any single extreme points likely the result of electronic noise. For both the CO_2_ and O_3_ sensors, voltage values were recorded to the SD card as ADC values. These voltages were used as is for the CO_2_ sensor, but for the O_3_ sensor they were converted to a normalized resistance prior to analysis [2,4,19]. Note, all of the datasets for Weeks 0, 1, and 2 are complete with the exception of the O_3_ data from Y-Pod N3, on which the O_3_ sensor appears to have malfunctioned. Thus, this data has been excluded from the analysis.

Sensor signals were converted to concentrations using field calibration, which involves: (1) co-location with high-quality reference instruments; (2) the development of a calibration model using the air quality sensor signals, environmental sensor signals, and trusted reference data as well as a technique such as multiple linear regression; and (3) the evaluation of that model and its application to testing or validation data. Ideally; the sensors are co-located before and after the field deployment to better facilitate corrections for drift. It is common to incorporate environmental parameters into these calibration models as low-cost sensors are often cross-sensitive to temperature, humidity, and sometimes other pollutants [6]. This method of sensor quantification has been used by our research group as well as others [2,20,21] and with techniques such as linear regression, multiple linear regression, and machine learning [5,15]. Details of the calibration employed here are presented below.

#### 2.2.1. Quantification of CO_2_ Sensors

For CO_2_ sensor quantification, the Y-Pods were twice co-located with a LI-840A (Licor, Lincoln, NE, USA) placed at a regulatory monitoring site near downtown Los Angeles. The Licor LI-840A has an expected uncertainty of <1% of the reading as stated by the manufacturer, and the instrument is calibrated using a zero and two-point span calibration with gas standards. The Licor used in this study was calibrated prior to a deployment during the previous summer and was stored between these deployments. As a result of the time lag, we expect drift to have impacted the CO_2_ reference data. However, as we are interested in sensor to sensor comparisons and the sensor data is baseline shifted (as described below), this drift is of minimal concern. These two co-locations with the Licor were 8 weeks apart and included 17 days total, 12 of which were used for calibration model training and 5 of which were used for model testing. In this instance more of the co-location data was designated for training in order to increase the robustness of the model and expand the environmental conditions for which the model was trained. The model used, Equation (1), included predictors for temperature (Temp), absolute humidity (AH), time (t), and the sensor signal or voltage (V) and solves for the CO_2_ concentration (C):

C = (p_1_ + p_3_ * Temp + p_4_ * AH + p_5_ * t − V) * (−1/p_2_) (1)

Due to logistics and a lack of available reference data, both calibration co-locations occurred prior to the building-scale variability study (Figure 2). For this reason, further signal processing was necessary. Given that the CO_2_ calibration model is extrapolating in time, additional drift was expected. For this reason, the CO_2_ data was converted using the calibration model and then this data was baseline corrected (to remove drift), and finally the 10th percentile value from each Y-Pod was normalized to 400 ppm. We selected 400 ppm as it is the approximate atmospheric background concentration of CO_2_ [22]. In light of the goals of this case study–comparing relative differences across co-located verses deployed sensors–this additional processing was deemed reasonable. Furthermore, the results illustrate the high correlation and agreement between co-located sensors post-processing as would be expected and is also present in the calibration data (Appendix A).

#### 2.2.2. Quantification of the O_3_ Sensors

For O_3_ sensor quantification, the Y-Pods were co-located with API/Teledyne 400 instruments (San Diego, CA, USA) at two different regulatory monitoring sites. The first site was in Los Angeles in a mixed-use area with some nearby housing and industry. The second site was outside of Los Angles in Shafter, a rural Californian community. These two co-locations occurred prior to and following the building-scale field deployment and therefore no additional signal processing was necessary. The model, Equation (2), used included predictors for temperature (Temp), absolute humidity (AH), time (t), the normalized sensor resistance (R/R_0_), as well as an interaction term between temperature and concentration, and solves for the O_3_ concentration (C). The interaction term is intended to address not only changes in baseline driven by temperature but changes in the magnitude of sensor response driven by temperature. This model has been demonstrated as well performing for this sensor in previous studies [2,16]:

C = (p_1_ + p_3_ * T + p_4_ * AH + p_5_ * t − R/R0) * (−1/(p_2_ + p_6_ * T)) (2)

## 3. Results and Discussion

### 3.1. Field Calibration Results (Sensor System Uncertainty)

Table 1 below provides the performance statistics from the generation and validation of the calibration models. The complete statistics for individual Y-Pods as well as time series data are available in Appendix A. For both CO_2_ and O_3_, there is relative consistency across the training and testing datasets. Additionally, the RMSE for the O_3_ sensor was consistent with uncertainty typically cited for both this same sensor and other metal oxide O_3_ sensors [2,16,23]. A previous study using the CO_2_ sensor in a portable sensor system found a RMSE ranging from approximately 9–16 ppm depending on the calibration model selected [4].

### 3.2. Neighborhood-Scale Variability

**Figure 3:**
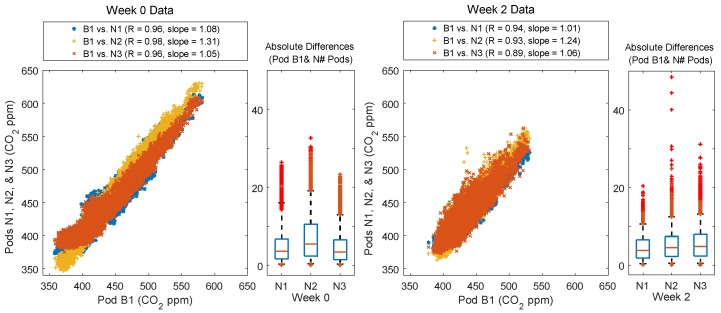
Scatter plots showing each neighborhood Y-Pod (N1, N2, and N3) vs. Y-Pod B1 for Weeks 0 and 2. The boxplots show the absolute differences between B1 and each of the neighborhood pods, with the whiskers at the 5th and 95th percentile respectively.

Comparing Week 0 (co-located) to Week 2 (deployed to field sites), there is increased variability in both the CO_2_ and O_3_ data. For CO_2_, this variability is most extreme in the comparison between B1 and N3, which was also the site furthest away from B1 and closest to the highways. For this pair of sensors, the correlation decreases from 0.96 to 0.89 and the spread in the absolute differences as well as the median absolute difference increases, see Figure 3. This is not the case for the comparisons of B1 to N1/N2 where there is only a very small decrease in correlation. Examining the time series plots (available in Appendix B) reveals differences in the variability seen in Week 0 versus Week 2. For Week 0 the variability seems primarily driven by offsets in which one Pod is biased low or high for a period, whereas for Week 2, the variability seems driven by differences in trends between the sites typically in the form of short-term enhancements. These enhancements present in the Week 2 data are likely sources or plumes impacting the sites unevenly.

**Figure 4:**
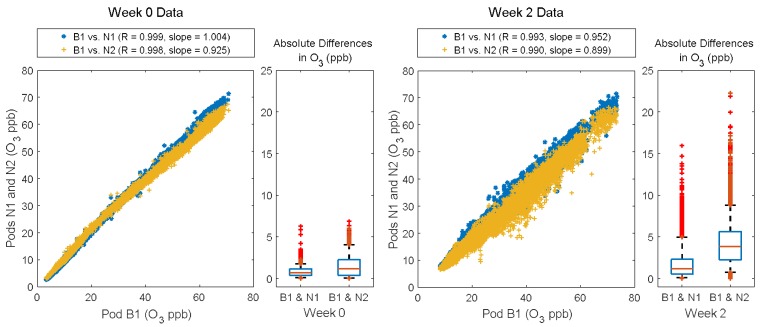
Scatter plots showing each neighborhood Y-Pod (N1, and N2) vs. Y-Pod B1 for Weeks 0 and 2. The boxplots show the absolute differences between B1 and each of the neighborhood pods, with the whiskers at the 5th and 95th percentile respectively. The ozone sensor for N3 malfunctioned and the data was not included.

For O_3_, spatial variability across field sites was much more apparent. Although there was little change in the correlation coefficient, there was an increase in the spread in both the scatterplot and the boxplot (Figure 4). For Week 0, nearly all the absolute differences between B1 and N1/N2 were below the expected uncertainty (RMSE = 5.28 ppb). For Week 2, after the Y-Pods were spatially deployed the spread increased to well above the RMSE, see Figure 4. The time series plots (Appendix B) confirmed that this increased variability was primarily driven by short-term dips in O_3_ likely caused by localized destruction occurring in a NO_x_ plume. While it is possible that the differences in increased variability between the sensor types were in part due to CO_2_ being a primary pollutant (thus less well-mixed) and O_3_ a secondary pollutant (generally more well-mixed), it is also worth noting that the CO_2_ sensor has a lower signal/noise than the O_3_ sensor in this application.

### 3.3. Building-Scale Variability

**Figure 5:**
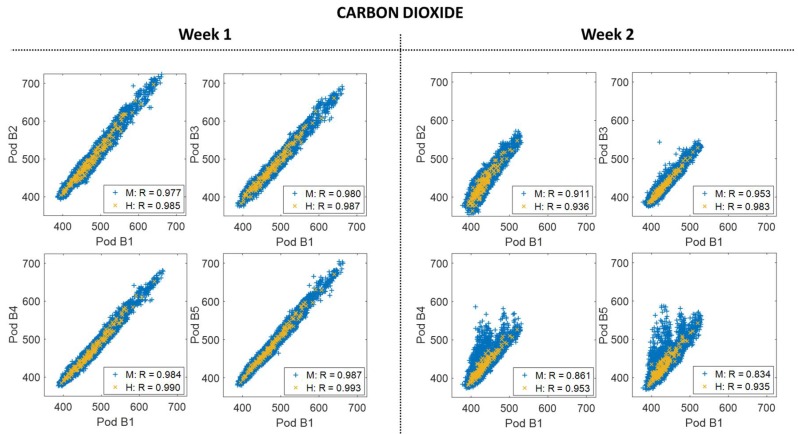
The scatter plots to the left show the correlation between Y-Pod B1 and each of the added building Y-Pods (B2, B3, B4, and B5) for both minute (M) and hourly (H) CO_2_ data for Week 1 (co-located). The scatter plots to the right show the same correlations, again with minute and hourly data, but for Week 2.

**Figure 6:**
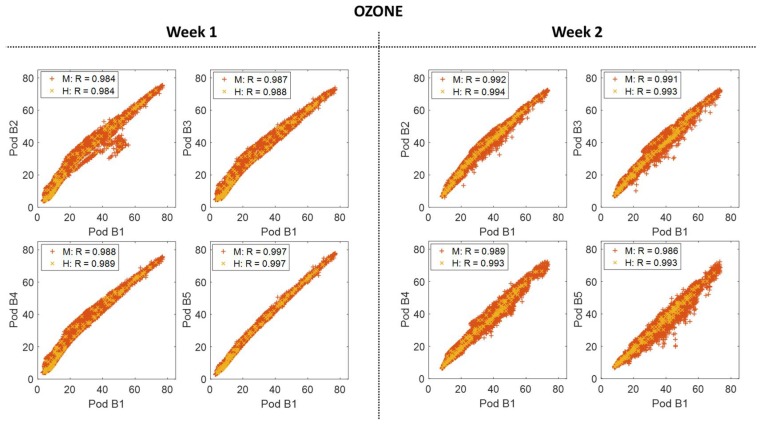
The scatter plots to the left show the correlation between Y-Pod B1 and each of the added building Y-Pods (B2, B3, B4, and B5) for both minute (M) and hourly (H) O_3_ data for Week 1 (co-located). The scatter plots to the right show the same correlations, again with minute and hourly data, for Week 2 when they were spatially deployed around the building site.

Somewhat surprisingly spatial variability was also observed at the building-level for both sensor types when comparing Week 1 (co-located at the building) and Week 2 (deployed). For CO_2_, there was a decrease in the correlations on the same scale as occurred across some of the neighborhood sites (Figure 5). For O_3_, again there are no significant changes to the statistics, but there is an increase in spread (Figure 6), similar to Figure 4. The time series (Appendix B) showed the events driving these differences were short-term in nature and appeared to be driven by local emissions or transported plumes. This influence of nearby emissions events was observed by Miskell and colleagues as well [10].

Hourly-averaged data was added to both Figure 5 and Figure 6 to determine whether this spatial variability impacted data on more typical temporal reporting scales. Similar to Miskell and colleagues, the variability does not seem to impact the hourly O_3_ data [10]. However, given the decreased correlation coefficients (particularly for sites B2 and B5), it appears there was some variability still present in the hourly-averaged CO_2_ data.

For both pollutants, the most dramatic differences were between sites B1 and B4/B5, the two sites at the back of the building. Speaking with community partners from the project we determined that the building has both a natural gas hot water heater and natural gas dryers toward the back of the building where there are also pipes that appear to be venting these emissions. Sources on the building would seem to explain the large magnitude of the observed variability. By comparison, for the sites B2 and B3, which were on the front of the building above the road, there were occasional increasing spikes for CO_2_ and decreasing spikes for O_3_ that are smaller in magnitude. The range of responses observed in the sensors, along with this contextual information affirms that multiple pollutant sources were impacting the building in an uneven manner.

**Figure 7:**
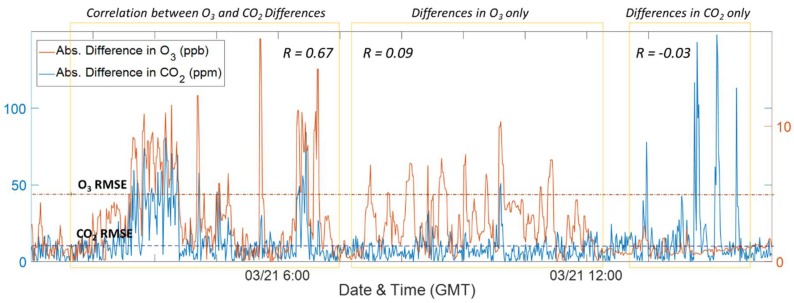
Time series of absolute differences between Y-Pod B1 and Y-Pod B5 for CO_2_ (blue) and O_3_ (red), the RMSE for both the CO_2_ and O_3_ sensors are indicated using dotted lines. The yellow boxes highlight periods where the differences in the two signals are well-correlated verses periods where the differences are occurring primarily in the CO_2_ or O_3_ signal. The correlation coefficient (R) has been added to contrast the different periods.

**Figure A6:**
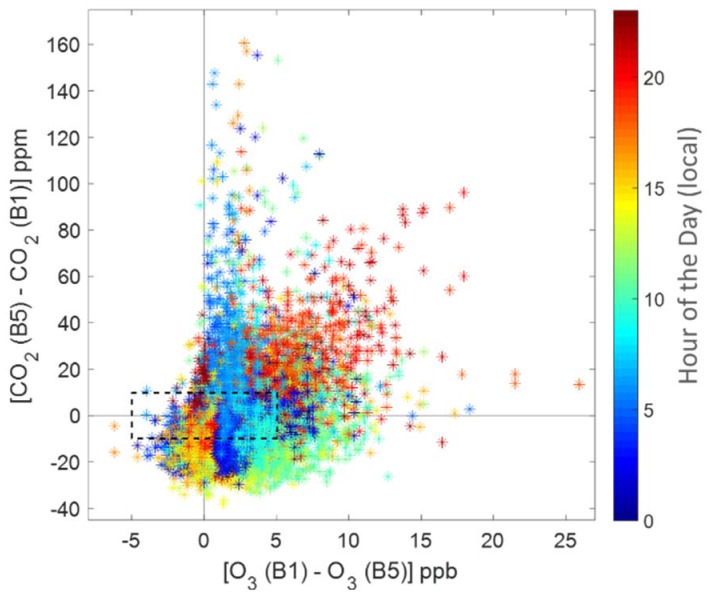
Scatter plot of differences between B1 and B5 (on the x-axis, for O_3_, B1–B5; and on the y-axis, for CO_2_, B5–B1), colored by the hour of the day. The dotted lines around the center axes indicate the respective RMSEs for CO_2_ and O_3_.

Providing further evidence for multiple sources, Figure 7 includes the absolute differences between Y-Pod B1 and B5 for CO_2_ (in blue) and O_3_ (in red). There are periods where the differences between CO_2_ and O_3_ were well-correlated indicating a shared source. Following this period were instances where the differences were primarily visible in one pollutant or the other. This lack of correlation likely indicates two separate sources, one with relatively more CO_2_ and another with more NO. Furthermore, there were many instances where these differences between the two building sites were well above the RMSE values. In Figure A6 (Appendix B) the spatial differences have been plotted in such a way as to highlight the temporal aspect of both the increases in CO_2_ and decreases in O_3_ at the B5 site. The correlation between differences in CO_2_ and O_3_ occur primarily in the evening hours, while the uncorrelated periods result in enhancements during early morning and daytime hours. These temporal patterns also point to separate sources influencing the sensor data.

**Figure 8:**
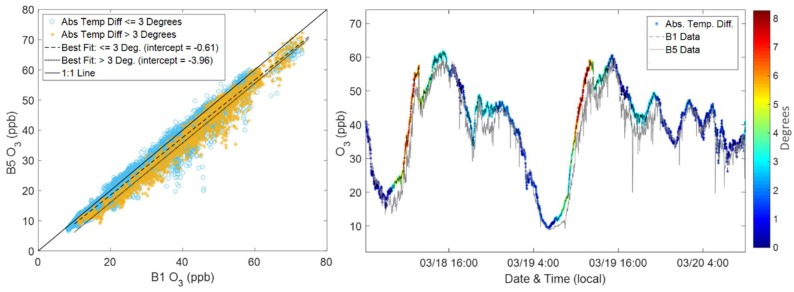
Two plots illustrating the effect of temperature differences between the pods. The scatter plot (**left**) depicts B1 vs. B5, separating points where the temperature difference between the two pods is less than and greater than three degrees Celsius. The time series (**right**), shows two days of data from B1 and B5 where the B1 data also has an overlay of temperature differences between the pods.

In addition to nearby emission events, Miskell and colleagues observed that direct sunlight causes thermal variations in the instruments causing variability [10]. We compared the internal temperatures in the Y-Pods to determine whether this could be a source of variability in our study as well. Figure 8 depicts the variability in light of temperature differences. Again, B1 was placed on a roof with no nearby obstructions meaning that it was exposed to more direct sun than B5, which was placed on a fire escape in an alley. In Figure 8, the internal temperature differences, between B1 and B5, less than three degrees Celsius were plotted separately from differences greater than three degrees Celsius. The line of best fit for the group with larger temperature differences (in yellow) illustrates a consistent bias in the data at low and high concentrations. This bias is visible in the time series as well, the B1 values are consistently greater than the B5 values when the temperature difference is above three degrees. Conversely, B1 and B5 are better matched in terms of long-term trends for smaller temperature differences. Although the calibration model does incorporate corrections for temperature effects, the model would be unable to account for the small differences driven by direct sunlight exposure as this would be difficult to control during co-location. The corrections incorporated into the calibration model are intended to deal with less acute temperature effects (e.g., diurnal patterns). 

Siting choices and additional shading for the sensor systems could reduce this variability. Although some of the variability between building-sites can be attributed to thermal differences, it is important to recall that this variability is displayed as a bias rather than the larger spread associated with the variability driven by nearby emissions. Therefore, this variability would be unlikely to affect any conclusions about spatial differences due to sources in the same way the short-term enhancements would when examining high temporal resolution data.

### 3.4. Impact of Siting Choices on Neighborhood Varibaility Analysis 

**Figure 9:**
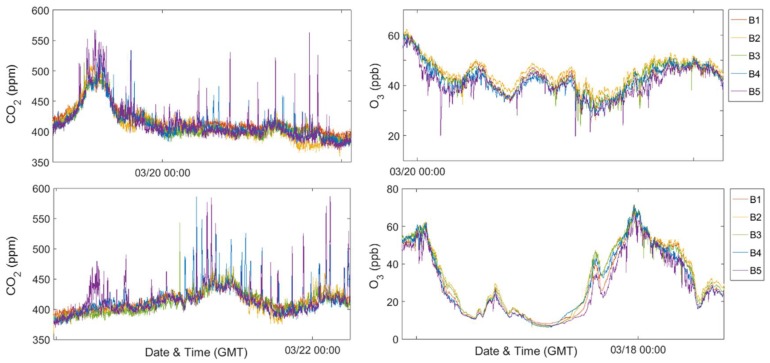
Time series of CO_2_ data (**top** and **bottom left**) and O_3_ data (**top** and **bottom right**), each showing approximately one-day of data from the building-sites during the Week 2 period.

In agreement with the findings of Miskell and colleagues, we have observed that local emissions or plumes can drive intra-site variability as well as temperature differences caused by exposure to direct sunlight [10]. Also, as with the previous study, this spatial variability does not impact O_3_ concentrations on typical reporting scales (hourly or eight-hour averages for example). However, the same is not necessarily true for CO_2_ suggesting it may be valuable to further investigate this aspect of variability for primary pollutants. The spatial variability observed here becomes especially important for communities interested in high-time resolution data, which may be used to assess exposure and/or understand the impact of local emission sources within a neighborhood. When high temporal and spatial resolution is of interest, incorrect placement could result in the inappropriate attribution of sensor responses or failing to record emissions that are present. Figure 9 includes several days of data demonstrating the large magnitude of differences that can be observed across a single site. 

**Figure 10:**
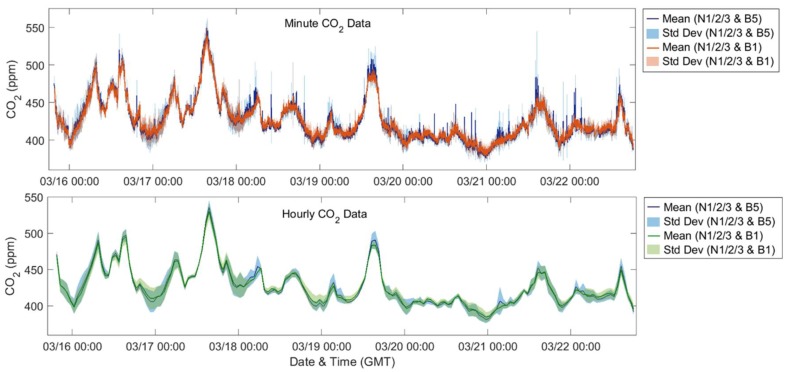
Time series of CO_2_ data (**top**: minute-median, **bottom**: hourly-averaged), showing the mean and standard deviation of different sets of Y-Pods.

**Figure 11:**
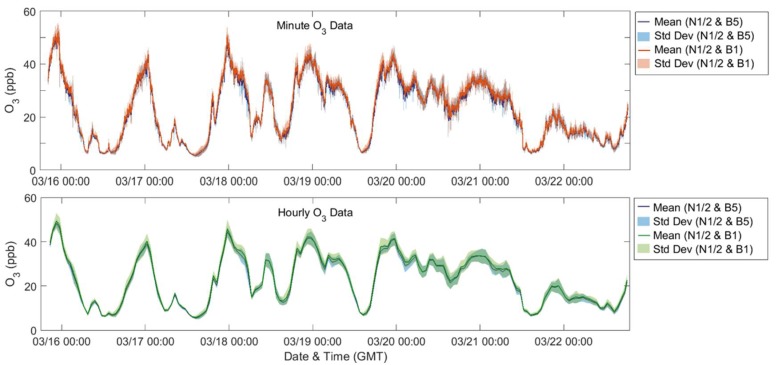
Time series of O_3_ data (**top**: minute-median, **bottom**: hourly-averaged), showing the mean and standard deviation of different sets of Y-Pods.

To further explore the impact of the building-scale variations on the community-scale spatial differences, Figure 10 and Figure 11 depict the average of the neighborhood sites with one building site selected and assumed to be representative for that location. The shading on the plot indicates the standard deviation for each mean. For the first case, in blue, Y-Pod B5 was selected as the building site Pod and for the second case, in red for minute median and green for hourly averaged data, B1 was selected. Similar to the previous comparison, there are minimal differences between the hourly O_3_ datasets and only a few instances in the hourly CO_2_ data where the mean of the B5 dataset differs beyond the standard deviation of the B1 dataset. However, examining the minute-median data for either pollutant, one might draw different conclusions regarding the neighborhood variability depending on which building site was selected. For example, one might anticipate more variability with B5 selected, or fewer local sources capable of scavenging O_3_ with B1 selected. If examining the maximum daily CO_2_ concentrations, the results for several days would differ. Regardless of which building site is selected, the diurnal trends are consistent potentially providing an indication of regional trends. Also, for the minute data, this difference between the datasets is more extreme for the CO_2_ data possibly due to CO_2_ being a primary pollutant and less well-mixed in the atmosphere.

### 3.5. Generalizability of Building-Scale Spatial Variability & Potential Recommendations

There are a few aspects of this study that limit generalizability: we used short periods of data, we only examined the variability around one building in Los Angeles (variability might look different around a different structure or in a different city), and the two sensors types we used rely on different operating principles. Given these limitations, there are still recommendations based on this analysis that can be made. As the following recommendations are intended for individuals or groups interested in conducting sensor studies, more general “best practice” recommendations have been included as well. While some of these are more general, specifically the first and fourth ones, the results of the study nonetheless affirm their value. Furthermore, these suggestions complement the US EPA’s existing recommendations for planning a study and siting sensors [11]. These recommendations are especially relevant to studies involving high-time resolution data on a neighborhood or source-scale:

- *Compare Sensors*: Co-locating sensors in the field will support a better understanding of inter-sensor variability prior to their deployment, which will aid in attributing new variability introduced by the deployment of sensor systems to separate sites. These relative comparisons can also be valuable if there are problems with the calibration.
- *Placement and Distribution*: To study a particular emission source, place sensors upwind and downwind of the site of interest, at varying distances. Some of the sensors should have a line of sight to the emission source. Consider factors such as typical wind directions and potential obstructions, which may impact the transport of emissions. These placements should also minimize added variability when possible. For example, shading all sensor systems, placing them on the same sides of buildings, or placing them exclusively on rooftops could reduce the variability and biases that result from occasional direct sunlight.
- *Supplementary Materials Sensor Data*: Consider using multiple systems or sensor types. The ability of sensors to capture variability on small spatial scales could be leveraged to aid in source identification by placing multiple sensor systems at a site with the objective of capturing local emissions with some systems and targeting exclusively regional trends with other systems. Leveraging data from multiple sensor types could also shed light on sources and emissions by studying the correlations or temporal patterns of data from sensors intended to measure different target pollutants.
- *Document Deployment*: Document your deployment in writing and with photos (take photos of the sensor systems from different angles and photos from the sensors of what they “see”). Learning about nearby activities could provide contextual information that can aid in data interpretation and reduce the misinterpretation of sensor data.

## 4. Conclusions

This deployment demonstrated how the variability in CO_2_ and O_3_, measured using low-cost sensors, across a single sampling site can be comparable to the variability across several sites in a neighborhood. However, this spatial variability occurs primarily in high-time resolution (<1 h) data as it seems to be driven by nearby emission plumes and occasional thermal differences. As Miskell and colleagues reported these differences do not persist at typical reporting scales [10], but if a researcher or community is interested in high-temporal resolution data then this variability could become significant. This variability might also be more important to consider for studies taking place on smaller spatial scales, such as the neighborhood scale at which this study takes place, rather than larger regional scales.

While minute-level data is not currently utilized for regulatory purposes, this level of data can provide powerful preliminary and Supplementary Materials information when it comes to understanding the activities and experiences in a community and at local scales. Furthermore, the presence of building-level variability does not exclude sensors from being used in air quality investigations, but rather affirms their ability to detect these differences in trends. Through attention to siting and thorough planning/documentation, there is the potential for the collection of an entirely new type of data that could for example, inform detailed investigations into the impact of a single source on a neighborhood, track the transport of emissions through an area, or clarify the acute effects of brief, high-concentration exposures. These potential applications suggest that this new type of data, made possible by sensors, could eventually support improved public health.

## Figures and Tables

**Table 1:** Performance Statistics as Compared to Reference Datasets (Averaged for all Y-Pods).

	Statistic	Training	Testing
**CO_2_**	*R^2^*	0.92 (0.03)	0.89 (0.06)
	RMSE (ppm)	8.33 (1.71)	10.09 (3.16)
	MB (ppm)	−0.02 (0.02)	3.89 (5.95)
**O_3_**	*R^2^*	0.97 (0.01)	0.94 (0.02)
	RMSE (ppb)	3.65 (0.42)	5.28 (0.86)
	MB (ppb)	−0.09 (0.02)	−2.30 (0.79)

*R*^2^—coefficient of determination, RMSE—root mean squared error, MB—mean bias, with standard deviations in parentheses.

**Figure A1:**
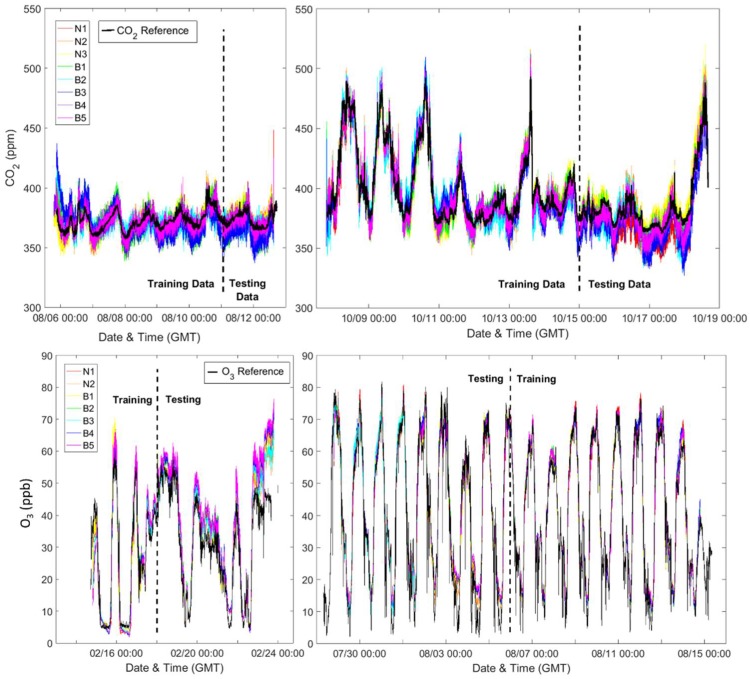
Time series of Y-Pod data (shown in colors), and reference data (shown in black), for CO_2_ (**top**) and O_3_ (**bottom**).

**Figure A2:**
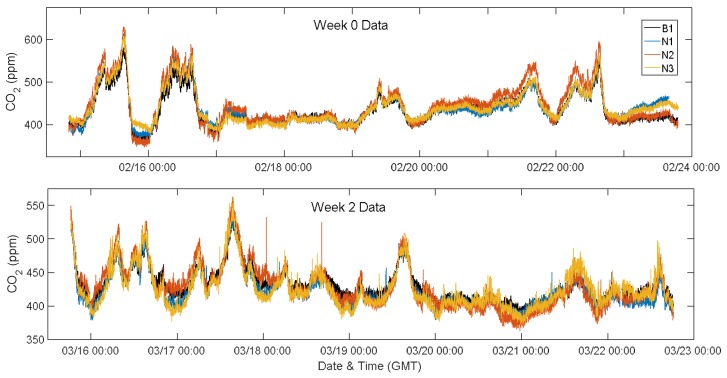
Time series of co-located (**top**) and deployed (**bottom**) Y-Pods, for examining neighborhood-scale variability of CO_2_.

**Figure A3:**
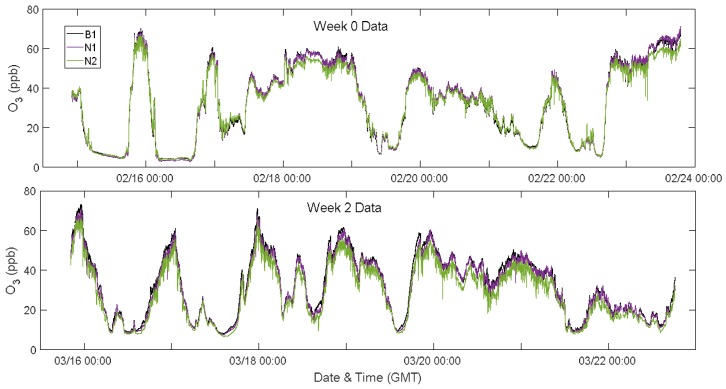
Time series of co-located (**top**) and deployed (**bottom**) Y-Pods, for examining neighborhood-scale variability of O_3_.

**Figure A4:**
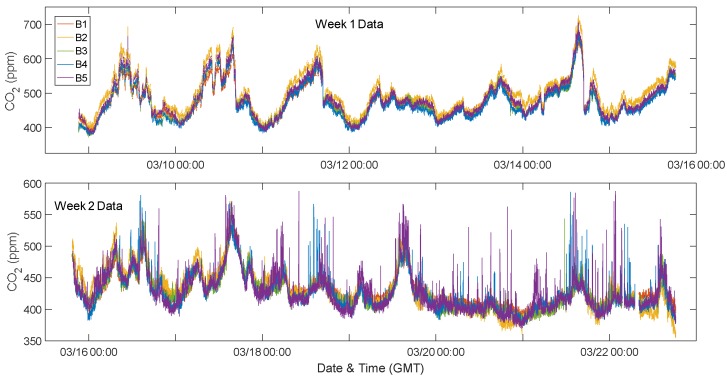
Time series of co-located (**top**) and deployed (**bottom**) Y-Pods, for examining building-scale variability of CO_2_.

**Figure A5:**
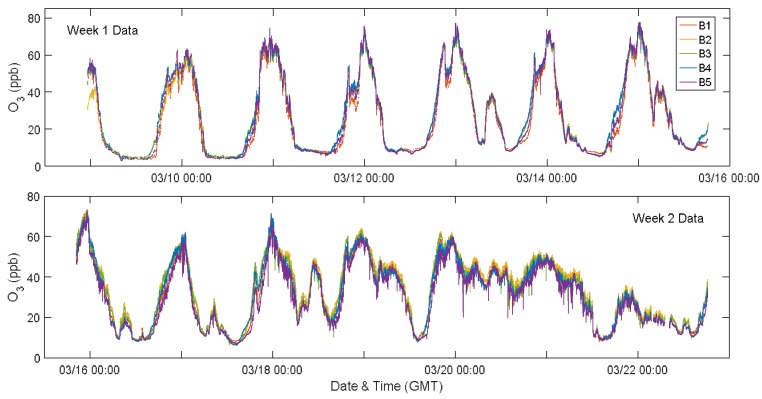
Time series of co-located (**top**) and deployed (**bottom**) Y-Pods, for examining building-scale variability of O_3_.

## Supplementary Materials

Processed sensor data is available by request, please contact the corresponding author. To discuss the availability of raw sensor data and associated code for processing, please also contact the corresponding author.

## Appendix A. Sensor Quantification Results

**Table A1:** Performance Statistics for each Y-Pod; RMSE—CO_2_ (ppm) & O_3_ (ppb); MB—Mean Bias.

Carbon Dioxide							Ozone					
	Training Data			Testing Data			Training Data			Testing Data		
Pod	*R* ^2^	RMSE	MB	*R* ^2^	RMSE	MB	*R* ^2^	RMSE	MB	*R* ^2^	RMSE	MB
B2	0.93	7.32	−0.01	0.94	9.69	−6.35	NA	NA	NA	NA	NA	NA
B3	0.93	7.85	−0.04	0.93	7.39	2.69	0.97	3.45	−0.11	0.94	5.16	−1.88
B4	0.86	11.2	0.01	0.83	11.3	5.84	0.96	3.88	−0.07	0.96	4.73	−2.18
B5	0.94	7.35	−0.03	0.92	7.25	1.90	0.96	4.00	−0.11	0.92	6.51	−3.07
B7	0.94	7.32	0.01	0.77	14.7	10.4	0.97	3.71	−0.13	0.94	5.30	−1.99
B8	0.91	9.11	−0.04	0.88	9.00	0.39	0.98	2.78	−0.08	0.97	3.96	−0.94
C9	0.88	10.3	−0.04	0.89	14.6	12.8	0.97	3.74	−0.08	0.95	5.10	−2.96
D2	0.95	6.19	−0.02	0.93	6.83	3.49	0.96	3.95	−0.08	0.93	6.21	−3.08
Ave	0.92	8.33	−0.02	0.89	10.1	3.89	0.97	3.65	−0.09	0.94	5.28	−2.30
SD	0.03	1.71	0.02	0.06	3.16	5.95	0.01	0.42	0.02	0.02	0.86	0.79
